# Description of swine producer biosecurity planning for foreign animal disease preparedness using the Secure Pork Supply framework

**DOI:** 10.3389/fvets.2024.1380623

**Published:** 2024-04-26

**Authors:** Magnus R. Campler, Marissa Hall, Kelsey Mills, Jason A. Galvis, Gustavo Machado, Andreia G. Arruda

**Affiliations:** ^1^Department of Veterinary Preventive Medicine, The Ohio State University, Columbus, OH, United States; ^2^Department of Population Health and Pathobiology, College of Veterinary Medicine, North Carolina State University, Raleigh, NC, United States

**Keywords:** swine disease, review process, continuity of business, biosecurity planning, Secure Pork Supply

## Abstract

**Introduction:**

Preventing potential foreign animal diseases is a high priority, with re-emerging threats such as African Swine Fever emerging close to North American borders. The Secure Pork Supply (SPS) plan provides a voluntary framework for swine producer biosecurity planning and disease outbreak preparedness. However, biosecurity knowledge varies greatly among swine veterinarians, managers, and caretakers within the industry, which impacts the understanding, quality, implementation and biosecurity plan agreements with the SPS guidelines unless review procedures and quality control mechanisms are in place. Therefore, this study aimed to describe and identify the level of biosecurity planning agreements between producer-and/or swine veterinarian-made biosecurity plans for commercial swine sites and the SPS plan guidelines during a review process.

**Material and methods:**

Biosecurity maps (*N* = 368) and written plans (*N* = 247) were obtained from six Midwest swine companies/veterinary clinics. Maps were evaluated on accuracy and placement of mandatory map features based on SPS guidelines, and discrepancies between the development of producer-made biosecurity maps and written biosecurity plans. Multivariable mixed logistic regression analyses were conducted to identify differences in SPS planning accuracy based on herd size, production stage, and characteristics related to geographical site location (e.g., land cover type and expected feral swine population density in the region).

**Results:**

In this study, 55.8% (205/368) of all provided biosecurity maps had to be revised due to misplaced or missing map features. In addition, 80.9% (200/247) of the written plans had one or more conflicts with the corresponding biosecurity maps. The main biosecurity planning issues involved feed delivery activities, where the mapping of vehicle movements (89.9%, 222/247) were in direct conflict with the written SPS plans. Sites located in areas with a moderate expected feral swine population density had 3-fold increased odds of needing map revisions compared to sites with low expected feral swine population density. Sites located in predominately farmland had 7.3% lower odds of having biosecurity map and SPS plan conflicts for every 1.0% increase in farmland landcover in a 10-km radius around the swine site.

**Discussion:**

Human oversight or lack of knowledge regarding biosecurity planning and implementation is common, which may culminate in important preparedness shortcomings in disease prevention and control strategies for U.S. swine farms. Future efforts should focus on additional biosecurity training for swine producers and veterinarians alongside with quality control benchmarking of producer made plans.

## Introduction

The Secure Pork Supply (SPS) platform (https://www.securepork.org) aims to ensure a comprehensive and standardized continuity of business plan for the U.S swine industry in the event of a foreign animal disease outbreak, such as Foot-and-Mouth disease (FMD), Classical Swine Fever (CSF) or African Swine Fever (ASF) (1). Created through collaborative efforts between the swine industry, state and federal government officials, and University members (University of Minnesota and Iowa State University) and funded through the U.S. Department of Agriculture, Animal and Plant Health Inspection Service (USDA APHIS) and the National Pork Board, it provides swine producers a voluntary opportunity to access material and guidance in how to improve the level of biosecurity throughout their production systems. The SPS framework allows swine producers to create biosecurity maps and enhanced preparedness plans detailing the plan of action for each site during foreign disease outbreaks. Having readily available preparedness plans and biosecurity maps can improve participating swine producers' position to move animals to market during periods of partial industry lockdowns due to disease outbreaks or supply chain disruptions as seen during COVID-19 [e.g., (2–4)]. Although the SPS platform provides comprehensive training videos and reading materials online, digesting and absorbing the provided information may be overwhelming and time consuming to both swine producers and swine veterinarians. The understanding, implementation and execution of biosecurity maps and plans needs to be high for the biosecurity benefits of these plans to be maximized (1). It has been previously reported that producers are more likely to implement biosecurity measures after reported outbreaks, such as swine influenza (5) or porcine reproductive and respiratory syndrome virus (PRRSV) (6) have already occurred. Thus, a combination of limited biosecurity understanding and planning issues may result in less-than-optimal executions and implementations of SPS plans; which would result in delayed responses to real world scenarios and complicate disease containment and eradication if a pathogen is introduced into the U.S. Complete biosecurity plans and associated data also allow for improved risk modeling for disease spread (7, 8). Therefore, including data for factors that may facilitate the spread of disease is crucial to improve risk modeling and subsequent risk management. For instance, the inclusion of landcover compositions and feral swine population densities (9, 10) around study sites or areas in risk modeling have shown to be important predictors for, but not limited to, spread of pathogens such as ASF (7, 11, 12), PRRSV (8, 13–15), and pseudorabies (16). Thus, facilitating improved biosecurity knowledge and accurate implementation of producer-/veterinarian-made biosecurity plans are crucial to ensure that producers are prepared for existing local threats such as feral pig-derived pathogens or novel foreign animal diseases (FADs). Furthermore, a comprehensive understanding of both the local and regional landscape help health officials to effectively update existing models on disease risks and spread and to make key response decisions in case of FADs that threaten the U.S swine industry.

The objectives of this study were to (1) describe and identify biosecurity planning agreements and deviations from SPS guidelines in producer-and/or swine veterinarian made biosecurity plans for commercial swine producers in the Midwest during a review process, (2) investigate factors of interest including site size, landcover and local feral pig density on the occurrence of said biosecurity agreements and deviations. We hypothesized that the amount of biosecurity agreements and deviations from SPS guidelines would be different depending on swine site size, landcover, and local feral swine density.

## Materials and methods

### Secure Pork Supply guidelines, and biosecurity map and enhanced biosecurity plan development

**Figure 1:**
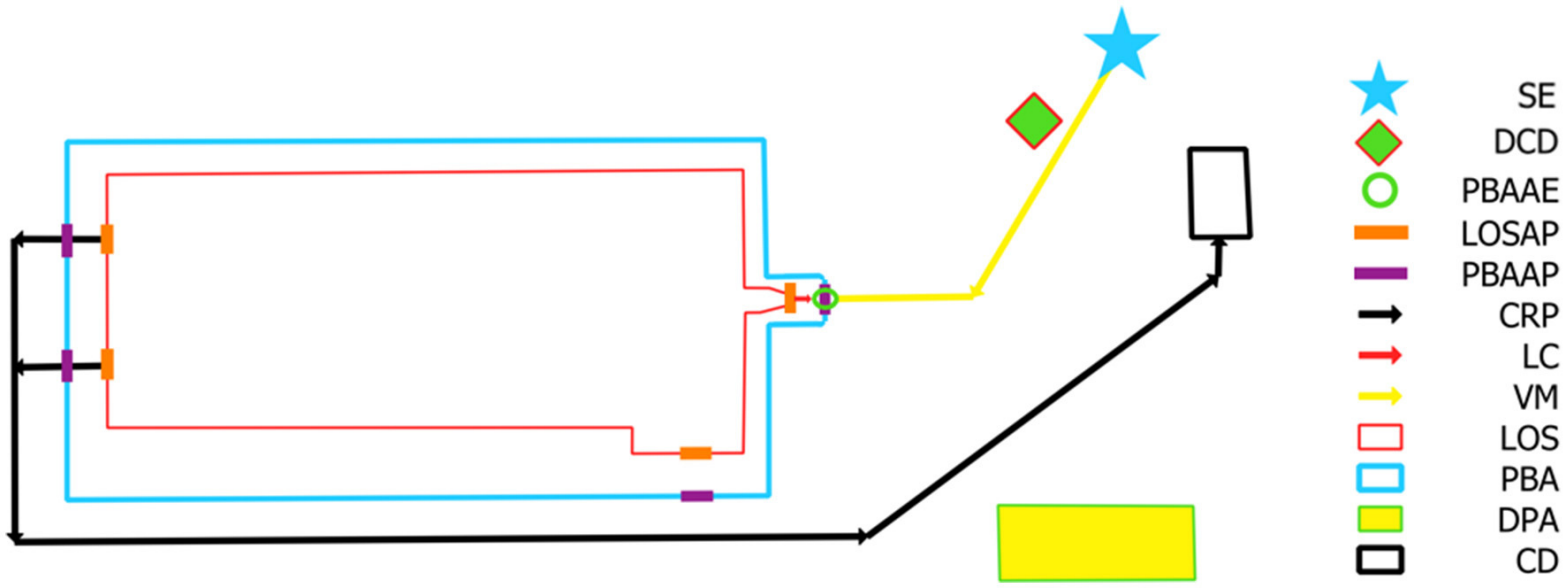
Outline of a Secure Pork Supply biosecurity map for a Wean-to-finisher site with each of the required map features and associated legend based on RABapp™ standards (17). SE, site entry; DCD, designated cleaning and disinfection area; PBAAE, perimeter buffer area access entry for animals only; LOSAP, line of separation access point; PBAAP, perimeter buffer area access point; CRP, carcass removal path; LC, loading chute; VM, vehicle movements; LOS, line of separation; PBA, perimeter buffer area; DPA, designated parking area; CD, carcass disposal location.

Swine farm managers, swine veterinarians and swine biosecurity managers in the Midwest were invited to participate in the SPS program and plan review through workshops, pork associations and investigator's professional network contacts. Data sharing agreements, data gathering and storage as well as contract signing for all participants were facilitated and hosted through a third-party pork association and stored on their two-factor authentication (2FA) cloud servers using Microsoft Teams (Microsoft Teams^®^, Microsoft Corporation, 2023) for protection of sensitive information. In total, 368 swine sites across six production systems opted for enrollment in the study. All producer data was temporarily made accessible to the research team throughout the duration of the project. All participation was voluntary, with the long-term aim going beyond the scope of the study and encompassing the attempt to improve site's eligibility with regulatory bodies to move pigs during FAD outbreaks under the SPS plan for continuity of business. All participants were tasked with creating outlined site biosecurity maps containing biosecurity measures and restricted movements which would be in place in case of FADs, following the SPS criteria (https://www.securepork.org/pork-producers/biosecurity/). All producer-made maps were professionally assessed, digitized, and mapped by project investigators using geographic information system software (QGIS 3.22.16, QGIS Geographic Information System. Open Source Geospatial Foundation Project http://qgis.org). Each site assessment encompassed the completeness of the map, the correct placement of specific map features, the functionality of the complete map (including accessibility and practicality for farm movements of vehicles, live pig transports, and removal of deadstock) as well as its agreement with a site-specific written SPS plan. The SPS criteria included twelve features to be placed and implemented correctly on the biosecurity map (Figure 1). Each biosecurity map, where applicable, included a site entry point (SE), a disinfection station (DCD), a perimeter buffer area (PBA) to outline the site area, lines of separation (LOS) to distinguish between structures housing pigs or used by swine caretakers, and other adjacent or auxiliary buildings structures not holding pigs on the site. In addition, all access points to be used during a disease outbreak had to be marked on the PBA (PBAAP) and LOS (LOSAP) outlines. Designated access points solely used for pigs such as loading chutes or designated loading docks (PBAAE) had to be marked for sites where it was specified that animal transports never entered the PBA for offloading of pigs. Finally, each loading area to be used during a disease outbreak (LC), each carcass removal path (CRP) and associated final or temporary carcass disposal area (CD), as well as all vehicle movements on the site (VM), and a designated parking areas (DPA) had to be placed on the biosecurity map (Figure 1). The comprehensive written SPS plan included site demographics, a detailed biosecurity implementation plan corresponding to the biosecurity map layout, farm routines, and contact information (Supplementary Presentation 1). For each site, the written SPS plans provided were reviewed and compared to the respective site map for agreements and inaccuracies between the two.

### Biosecurity map and written plan compliance evaluation

Compliance to the SPS framework was checked using the Rapid Access Biosecurity (RAB) app (RABapp™) (17). Briefly, the RABapp™ website-based application is in both industry and government sectors across 21 states serves as a platform for standardizing the approval of biosecurity plans and storing quality movement data. The RABapp™, is designed for use by State Animal Health Officials (SAHOs) and swine industry veterinarians. Through a user-interface, the RABapp™ provides stakeholders with a secure mechanism for uploading standardized biosecurity plans, which is subsequently processed through rigorous quality control procedures before is routed for SAHOs to undergo revision and approval (17).

For the purpose of the project, each swine site was evaluated based on two main criteria, (a) biosecurity map feature placement and implementation, and (b) consistency in implementation between biosecurity maps and SPS plans. Each biosecurity map was evaluated on whether all the mandatory features were placed on the map and if the placement of each of the map features were in line with the SPS plan and biosecurity guidelines (Table 1). For instance, if a designated carcass disposal area was present on the biosecurity map but simultaneously placed in front of an animal loading area, or next to a feed bin, the biosecurity map would not follow SPS guidelines due to failure to provide accessibility for incoming animal- or feed transport vehicles, and for breaching biosecurity guidelines regarding proper management of deadstock. Each biosecurity map could disagree with the guidelines for one or multiple issues, but the number of individual violations within each map feature category was not quantified. In addition, each biosecurity map was compared to the written SPS plan provided together with the biosecurity map for consistency as well as any written discrepancies from what was depicted in the biosecurity map. For example, if the SPS plan stated that all feed bins would be filled from the outside of the PBA using an auger and no feed trucks were allowed to physically enter the site, but the biosecurity map had multiple feed bin locations both outside and inside the provided PBA and out of range of the feed trucks filling capabilities, this would be considered a discrepancy. Therefore, if the SPS plan did not accurately reflect what was depicted in the biosecurity map or vice versa, the issues were noted, and the site did not pass the initial review. If the biosecurity map and SPS plan agreed, but the provided biosecurity implementation did not follow the SPS guidelines (either within the map or written plan), the site did not pass the initial review.

**Table 1:** Proportion (*n*/*N*) and percentage of missing and misplaced biosecurity map features, maps in need of revision, and in conflict with Secure Pork Supply (SPS) guidelines during an initial review of biosecurity maps for commercial swine sites (*N* = 368) within six seine companies/veterinary clinics in the Midwest.

**Statement**	**Map feature assessed**	***n*/*N* (%) of maps**
Biosecurity map missing a designated site entry point	SE	0/368 (0)
Biosecurity map missing a perimeter buffer area	PBA	0/368 (0)
Biosecurity map missing a line of separation	LOS	0/368 (0)
Biosecurity map missing designated loading areas	LC	103/368 (28.3)
Biosecurity map missing designated access points	PBAAP, LOSAP	0/368(0)
Biosecurity map missing a carcass disposal area	CD	43/368 (11.7)
Biosecurity map missing a marked carcass removal path	CRP	27/368 (7.3)
Biosecurity map missing designated access points used for animals only	PBAAE	367/368 (99.6)
Biosecurity map missing a disinfection station	DCD	58/368 (15.8)
Biosecurity map missing a designated parking area	DPA	0/368 (0)
Biosecurity map missing designated vehicle movement routes	VM	0/368 (0)
Misplaced line of separation	LOS	14/368 (3.8)
Misplaced perimeter buffer area	PBA	47/368 (12.8)
Misplaced parking	DPA	50/368 (13.7)
Misplaced access point	PBAAP, LOSAP	156/368 (42.5)
Misplaced disinfection station	DCD	124/368 (40.1)
Biosecurity map needs revision (missing/misplaced features)	All features	205/368 (55.8)
Biosecurity map in conflict with the SPS plan^a^	All features	200/247 (80.9)

SE, site entry; DCD, designated cleaning and disinfection area; PBAAE, perimeter buffer area access entry for animals only; LOSAP, line of separation access point; PBAAP, perimeter buffer area access point; CRP, carcass removal path; LC, loading chute; VM, vehicle movements; LOS, line of separation; PBA, perimeter buffer area; DPA, designated parking area; CD, carcass disposal location.

^a^An enhanced written plan was not provided for 121 sites.

### Landcover and feral swine density assessment

Using QGIS 3.22.16, all 368 swine site coordinates were plotted on a World Geodetic System (WGS84) projection. Using a publicly available landcover data set from the National Land Cover Data Base (NLCD 2021) obtained from the Multi-Resolution Land Characteristics (MRLC) Consortium and The U.S. Geological Survey (USGS) collaboration (https://www.mrlc.gov/), landcover data from a 10-km (6.2-mile) buffer radius surrounding each site was extracted. The chosen buffer radius was based on the largest surveillance zone width recommended by the USDA African Swine Fever Response Plan “The Red Book” (18). The spatial resolution of the NLCD was 30 m^2^ and contained 16 land cover types [Open water, Perennial ice and snow, Developed (open space), Developed (low intensity), Developed (medium intensity), Developed (high intensity), Barren land, Deciduous forest, Evergreen forest, Mixed forest, Shrub/Scrub, Grassland/herbaceous, Pasture/hay, Cultivated crops, Woody wetlands and Emergent herbaceous wetlands]. The developed land cover types represent urban environments.

An expected feral pig density data set was obtained from the U.S. Department of Agriculture, Animal and Plant Health Inspection Service, Center for Epidemiology and Animal Health, Veterinary Services, Fort Collins, Colorado. The expected feral pig density data used in this study was derived from the predictive models and approximations developed and described in Lewis et al. (10, 19). In brief, biotic and abiotic factors such as, but not limited to, landcover, land use, enhanced vegetation index (EVI), forest canopy cover, predation pressure, precipitation, humidity and temperature, were used in multiple linear regression models to predict feral pig distribution across both native and non-native ranges with the assumption made that the feral pig population had reached biological equilibrium. The outcome of the models provided expected feral pig densities based on local landscape factors and likelihood of the predictive distribution of feral pig populations. Additional details are described in Lewis et al. (10). All swine site coordinates were plotted in QGIS and a buffer area with a 10-km (6.2-mile) radius surrounding each swine site was created, as previously described, from which the average expected feral pig density estimation was extracted. The spatial resolution for the expected feral pig density data set was 1.0 km^2^, or the number of estimated feral pigs/km^2^. The expected feral pig population density ranges used in our study were based on previously set thresholds by (19), low = 0–2 pigs/km^2^; moderate= 3–5 pigs/km^2^, and high = >5 pigs/km^2^) for the contiguous U.S.

### Statistical analysis

All descriptive statistics and statistical modeling were conducted using Stata (StataCorp, 2023. Stata Statistical Software: Release 15. College Station, TX:StataCorp LLC). Due to the low number of participating gilt development units (GDU), isolation and boar stud sites in the study, these were all grouped into one “mixed” production type category leaving a total of five production type categories (Sow, Mixed, Nursery, Finisher and Wean-to-finisher) for analysis. Collinearity between variables was assessed using Spearman correlation coefficient with a cut-off value of 0.60. Remaining continuous variables were visually controlled for linearity and functional form using locally weighted regression plots. Variables not meeting this condition were categorized using the median values. Herd size was categorized as ≤ 2,700 or >2,700 pigs. Landcover types were merged into four categories (1) Urban = Developed (open space), Developed (low intensity), Developed (medium intensity), Developed (high intensity, (2) Farmland = Pasture/hay, Cultivated crops, Barren land, Shrub/Scrub, and Grassland/herbaceous, (3) Forested = Deciduous forest, Evergreen forest, Mixed forest, and (4) Water = Open water, Woody wetlands and Emergent herbaceous wetlands. Additionally, as few swine sites (*n* =3) were located in high expected feral pig density areas, the high and moderate density levels were merged into one, leaving a low- (*n* = 327) and a moderate (*n* = 31) expected feral swine density category.

Univariable mixed logistic regression models were built for each of the assessment statements listed in Table 1, except for statements for which missing biosecurity map features did not occur (i.e., SE, PBA, LOS, DPA, VM). The univariable mixed logistic regression models were built using a dichotomous dependent variable structure (yes = 1; no = 0) with associations with a *P*-value <0.20 moving forward for consideration in multivariable modeling, in a backwards stepwise manner. Finally, a multivariable mixed logistic regression model was built for each of the assessment statements considered from the univariable modeling with, production type (Sow, Mixed, Nursery, Finisher, Wean-to-finisher), herd size (<2,700, ≥2,700), landcover (Urban, Farmland, Forested, and Water), and expected feral pig density (low, moderate) as independent variables and company/veterinary clinic used as a random effect. Variables in the multivariable model were deemed confounders and only kept in the final model if they changed the value of any other model coefficients by >20%, regardless of statistical significance. Statistical significance was declared at *P* < 0.05, and tendencies was declared at 0.05 ≤ *P* < 0.10.

## Results

### Site population descriptives

Six swine companies/veterinary clinics of varying size totaling 368 sites with a mean (±SD) herd size of 2,659 (±1,408) pigs chose to participate in the study. Full details on production type and herd size distribution by company/clinic are presented in Table 2. The landcover proportion (mean ± SD) within a 10-km radius surrounding the swine sites consisted predominantly of farmland (80.4 ± 11.0%) followed by forested areas (9.3 ± 10.4%), urban areas (8.3 ± 3.1%), and water (1.2 ± 2.8%) (Table 3). Only 30.7% (133/368) of all swine sites had a designated urban area landcover within the 10-km buffer zone. The mean (± SD) distance to that closest urban area within the 10-km buffer zone was 7.8 (± 1.9) km. The mean (± SD) expected feral pig density for all sites was 1.5 (± 0.9) pigs/km^2^. 91.6% of sites were situated in a low expected feral pig density area with a mean of 1.2 (± 0.4) pigs/km^2^, while 8.4% of sites were situated in a moderate expected feral pig density area with a mean of 4.1 (± 0.7) pigs/km^2^.

**Table 2:** Descriptive count of participating swine sites (*N* = 368) per swine company/vet clinics (A–F) and production type with respective herd size estimates (median ± SD, min, max) for project participants.

	**Company**										
**Production type**	**A**	**B**	**C**	**D**	**E**	**F**	**Total**	**Median herd size**	**SD**	**Min**	**Max**
Boar Stud	1	1	0	0	0	0	2	1,300	1,697	100	2,500
Finisher	49	37	14	0	21	5	126	2,100	936	500	4,800
GDU	7	8	0	3	0	0	18	2,200	885	1,200	4,700
Isolation	0	14	0	0	0	0	14	700	1,326	120	5,500
Nursery	20	20	2	0	2	1	45	3,600	1,995	1,100	12,000
Sow	8	17	0	6	3	0	34	2,500	1,320	1,100	6,400
Wean-to-finisher	52	61	1	0	15	0	129	2,500	1,304	1,000	9,700
**Total**	137	158	17	9	41	6	368	2,400	1,408	100	12,000

**Table 3:** Landcover (Farmland, Urban area, Forest, and Water) proportions (%) (median SD) of participating swine sites (*N* = 368) in Ohio per production type category [Sow, Mixed (Boar Stud/Isolation/GDU), Nursery, Wean-to-finisher, and Finisher] within a 10-km radius.

	**Landcover category**			
**Production type**	**Farmland (**±**SD)**	**Urban area (**±**SD)**	**Forest (**±**SD)**	**Water (**±**SD)**
Sow	83.2 (13.4)	7.1 (4.5)	7.4 (12.3)	1.4 (1.7)
Mixed	82.3 (4.5)	7.4 (2.7)	6.5 (2.8)	2.3 (2.1)
Nursery	84.1 (7.1)	7.7 (2.3)	7.4 (8.7)	0.8 (1.8)
Wean-to-finisher	83.3 (12.3)	7.1 (3.4)	6.1 (11.8)	1.4 (3.6)
Finisher	83.8 (11.2)	7.8 (2.8)	6.8 (10.9)	1.0 (2.4)
**Total**	83.5 (11.0)	7.5 (3.2)	6.5 (10.4)	1.1 (2.8)

Due to the use of a circular buffer area on top of square raster data, all rows will not add up to 100%.

### Characterization of biosecurity agreements and deviations

Excluding the loading areas designated for animal use only (PBAAE) feature, which was lacking for all biosecurity maps except one, the overall number of biosecurity maps that needed revision due to at least one missing or misplaced feature was 205/368 (55.8%). The number of biosecurity maps that conflicted with the written SPS plan was 200/247 (80.9%). Please note that a lower number of sites were eligible for this subset analysis given an enhanced written plan was not provided for 121 (32.9%) sites. Sixteen sites (16/335, 4.8%) failed to provide a legible biosecurity map that was sufficient for proper revision. The main conflicts between biosecurity maps and written SPS plans were inconsistencies or conflicting information regarding PBAAE, and how feed and animal deliveries would occur during an FAD. Only one map (1/247, 0.4%) provided a PBAAE feature despite all written SPS plans stating these would be clearly indicated on the biosecurity map. For feed deliveries, a vast majority of written SPS plans (231/247, 93.5%) stated that feed delivery trucks would not be allowed to cross the PBA during a FAD. Out of the same SPS plans, 222/247 (89.9%) stated that feed bins were only allowed be filled from outside the PBA through an auguring process. However, 187/247 (75.7%) of the respective biosecurity maps had PBA's drawn that rendered feed bins inaccessible from the outside the PBA despite the use of an auger. Finally, 24/247 (9.7%) of the biosecurity maps had feed bins placed both inside and outside the PBA.

For animal deliveries, 50/247 (20.2%) of the SPS plans stated that trucks were allowed to cross into the PBA during an FAD; but from those, 16/50 (32.0%) written plans had contradictive statements that animal deliveries were not allowed to cross into the PBA. All sites placed the SE, PBA, LOS, PBAAP, LOSAP, DPA and VM features on their maps. A summary of the proportion of missing and misplaced biosecurity map features are summarized in Table 1.

Results from the multivariable logistic regression models indicated that herd size, production type and feral swine density were predictors for the need for biosecurity maps' revisions due to missing or misplaced map features (Table 4). Moreover, sites located in areas with a moderate expected feral swine density had a 3-fold increase in odds of needing revisions compared to sites located in areas with a low expected feral swine density (OR = 2.98, *P* = 0.031, Table 4). Nursery sites and larger sites had lower odds of needing revisions compared to sow sites (OR = 0.21, *P* = 0.03) and smaller sites (OR = 0.37, *P* = 0.029), respectively (Table 4).

**Table 4:** Final multivariable mixed logistic regression models for statements investigating predictors for commercial swine sites with (1) missing or misplaced biosecurity map features (yes/no), and (2) conflicting map and written biosecurity plans.

**Statement (yes/no)**	**Variable**	**Category**	**OR**	**SE**	**CI 95%**	***P*-value**
(1) Biosecurity map having missing or misplaced map features	Herd size	<2,700	Referent			
		>2,700	0.37	0.17	0.15–0.90	0.029
	Production type	Sow	Referent			
		Mixed^a^	0.41	0.24	0.13–1.31	0.132
		Nursery	0.09	0.06	0.02–0.36	0.001
		Wean-to-finisher	0.22	0.12	0.08–0.62	0.004
		Finisher	0.18	0.1	0.06–0.53	0.002
	Feral swine density	Low	Referent			
		Moderate	3	1.51	1.1–8.1	0.031
(2) Biosecurity map in conflict with written plan	Herd size	<2,700	Referent			
		>2,700	0.45	0.2	0.19–1.09	0.073
	Farmland landcover		0.93	0.03	0.87–0.99	0.023

OR, odds ratio; CI, confidence interval.

^a^Merged categories due to low site numbers: gilt development unit, isolation and boar stud sites.

Significant differences are defined as *P* < 0.05 and tendencies as 0.05 ≤ *P* < 0.1.

Herd size and production type were predictors for conflicts between the biosecurity maps and the written plan (Table 4). Furthermore, sites located in a land cover predominantly consisting of farmland had 7.3% lower odds of having a conflict between the biosecurity map and the SPS plan for every additional percent of farmland within a 10-km radius around the site (OR = 0.93, *P* = 0.023, Table 4).

For multivariable modeling on individual missing biosecurity map features (Table 1), only the final model for “missing designated loading chutes (LC)” had important predictors identified. The model indicated that all production types had lower odds of missing designated loading chutes on their biosecurity maps compared to sow sites [Mixed: OR = 0.11 (estimate) ± 0.06 (SE), 0.03–0.35 (95% CI), *P* < 0.001; Nursery: OR = 0.06 ± 0.03, 0.02–0.18, *P* < 0.001; (Wean-to-finisher: OR = 0.16 ± 0.07, 0.07–0.37, *P* < 0.001; Finisher: OR = 0.15 ± 0.07, 0.06–0.35, *P* < 0.001). A summary with statistically significant predictors and tendencies for misplaced features is shown in Table 5.

**Table 5:** Final multivariable mixed logistic regression models for statements investigating predictors for misplaced biosecurity map features (yes/no).

**Statement (yes/no)**	**Variable**	**Category**	**OR**	**SE**	**CI 95%**	***P*-value**
Misplaced line of separation	Herd size	<2,700	Referent			
		≥2,700	0.53	0.41	0.12–2.41	0.41
	Production type	Sow	Referent			
		Mixed^a^	0.59	0.56	0.09–3.80	0.577
		Nursery	1.0	0.93	0.16–6.16	0.995
		Wean-to-finisher	0.08	0.09	0.01–0.80	0.032
		Finisher	0.41	0.31	0.09–1.83	0.246
Misplaced perimeter buffer area	Herd size	<2,700	Referent			
		≥2,700	0.81	0.33	0.37–1.80	0.607
	Production type	Sow	Referent			
		Mixed^a^	0.21	0.15	0.05–0.83	0.026
		Nursery	0.33	0.21	0.09–1.14	0.08
		Wean-to-finisher	0.11	0.06	0.04–0.32	<0.001
		Finisher	0.09	0.05	0.03–0.29	<0.001
Misplaced designated parking area	Production type	Sow	Referent			
		Mixed^a^	0.22	0.15	0.05–0.85	0.029
		Nursery	0.32	0.18	0.09–0.97	0.044
		Wean-to-finisher	0.10	0.06	0.03–0.32	<0.001
		Finisher	0.09	0.05	0.03–0.29	<0.001
Misplaced access points	Herd size	<2,700	Referent			
		≥2,700	0.5	0.17	0.26–0.99	0.047
	Production type	Sow	Referent			
		Mixed^a^	0.38	0.22	0.12–1.19	0.097
		Nursery	0.27	0.16	0.08–0.88	0.03
		Wean-to-finisher	0.34	0.17	0.13–0.88	0.026
		Finisher	0.45	0.22	0.17–1.18	0.104
Misplaced disinfection station	Herd size	<2,700	Referent			
		≥2,700	0.2	0.09	0.08–0.49	<.001

OR, odds ratio; CI, confidence interval.

^a^Merged categories due to low site numbers: gilt development unit, isolation and boar stud sites.

Significant differences are defined as *P* < 0.05 and tendencies as 0.05 ≤ *P* < 0.1.

## Discussion

Numerous guidelines have been proposed for the development and implementation of biosecurity measures within swine farms, and a wealth of resources is available to swine producers and practitioners such as the USDA APHIS “*Protect our pigs*” ASF initiative (20), the Pork Checkoff Programs biosecurity training (https://porkcheckoff.org/pork-production-management/biosecurity/), the Pork Information Gateway biosecurity resources (https://porkgateway.org/resource/biosecurity-of-pigs-and-farm-security/) and the SPS plan for continuity of business (https://www.securepork.org/). Despite the ready availability of such guidelines, there is a paucity of evaluations assessing the quality of these plans which is labor and time consuming for both producers and animal health officials, particularly concerning their applicability to FADs. This study strategically leveraged the implementation of the SPS, a voluntary platform, to systematically investigate potential biosecurity agreements or inconsistencies arising when end-users apply the information for the specific purpose of FAD preparedness. Our study showed a notable lack of consistency in the design of these plans at the site level, with more than half of the plans scrutinized exhibiting deficiencies requiring revisions, primarily attributed to suboptimal placement of biosecurity features required by the SPS platform. Remarkably, over 80% of the plans were identified with discrepancies between the biosecurity map scheme and the comprehensive written plan. These discrepancies are of critical concern, as they have the potential to result in delays and unintentional disease spread during emergencies. The challenges identified in our study underscore the imperative for robust oversight and quality assurance protocols during the development of biosecurity plans intended for deployment in response to FAD suspicions or confirmations. The responsibility for such oversight remains ambiguous, prompting the need to delineate whether herd veterinarians, animal health officials, or other stakeholders should assume this crucial role. Notably, even though biosecurity appears to be an essential requirement during an emergency situation in the U.S. (21), variations in resources and training opportunities across states further complicate the allocation of this responsibility.

Moreover, a recent survey study on swine producer biosecurity attitudes reported a high variability in SPS Plan biosecurity adoption and implementation. The study also reported that the likelihood of specific biosecurity practices was determined by individual producers' risk tolerance and perception of its feasibility and benefit to their operation (1). The dangers of producers' self-determination of what biosecurity they deem “sufficient” or what may cause inconveniences to their operation may not only provide a sense of false security but also thwart state mitigation efforts during FAD outbreaks. The survey reported that producers that suffered PRRSV or porcine epidemic diarrhea virus (PEDV) outbreaks were more likely to encourage biosecurity adoption compared to producers that experienced no outbreaks (1).

The absence of a mandated framework, coupled with lack of standardized guidelines and accessible training resources (22), may impede the prioritization of intensive tasks associated with FAD preparedness in instances where the perceived threat of incursion is not imminent. It may also lead to high variability in biosecurity practices, which could complicate official response, as noted during an Emergency Preparedness Exercise conducted in Iowa (23). Therefore, additional efforts to motivate biosecurity implementation and preparedness in swine producers in lieu of lessons learned from disease impacts that may have been avoided should be encouraged. The RABapp™ consortium is an example of an effective platform to expedite the national standardization of biosecurity plans while also harmonizing entering of data by producer and individualized plan review electronically by state department of agricultures.

During the development of our project, even though the initial focus was on pre-review assessments, the study investigators invested supplementary efforts to guide producers in progressively refining these vital biosecurity plans. Through online consultations, real-time data sharing options, and secure revision mechanisms, we facilitated the seamless completion of tasks, ensuring alignment with established standards. Such supportive measures appeared to be integral considerations for future initiatives to uphold the quality of biosecurity plans. A recent publication reported effectiveness of an online educational website to improve biosecurity knowledge of swine producers and veterinarians, which could be another option for standardizing biosecurity plan-making (24). Moreover, we advocate for the involvement of decision-makers, such as state veterinarians and their official teams, in the review process prior to potential outbreaks, thereby fortifying the timely implementation of biosecurity measures in the face of emergent challenges.

Expected feral swine density in the area, herd size, and production type emerged as critical variables in our models influencing the accuracy of various biosecurity features. Notably, the observation that sites situated in regions characterized by moderate expected swine density exhibit increased odds of requiring biosecurity plan revisions in comparison to sites in areas of low anticipated feral swine density is a cause for concern. This is particularly noteworthy considering the well-established transmission of several swine diseases, including ASF, by wild pigs (25, 26). Consequently, sites located in regions with moderate feral swine density may face an elevated risk of infection through potential direct or indirect contact with these wild animals, which, combined with potential biosecurity gaps, could lead to disastrous consequences. Considering these findings, a strategic recommendation emerges to prioritize regions with elevated disease introduction risk, for plan revisions. This proactive approach aligns with the overarching goal of enhancing the nation's preparedness for potential disease incursions, especially in areas where the threat of transmission from feral swine is heightened. Consistently, our analysis indicated that sow sites and sites with a larger animal population exhibit higher degree of challenges in the placement of biosecurity features on maps compared to other production types and smaller sites, respectively. Furthermore, these sites also appeared to have more inconsistencies when comparing the biosecurity map and corresponding written plans. We attribute this observation to the heightened complexity of these sites, necessitating meticulous planning and the consideration of numerous features. The significance of this finding lies in the potential “high consequence” associated with them when it comes to disease spread. Sow sites and larger facilities, by their increased production scale and service intensity, present more opportunities for unintentional pathogen transmission to other sites or agricultural facilities (27). Consequently, there is a critical need to prioritize the review of biosecurity plans for these high-consequence sites. Interestingly, this finding deviates from the common assumption that sow sites and larger facilities receive more attention regarding biosecurity measures (28). Our results suggest that despite the prevailing focus on these sites, there is still room for improvement and increased scrutiny in the planning and implementation of biosecurity measures to address the unique challenges posed by their complexity.

One of the strengths of this study is that individual site managers and veterinarians created their plans separately within each company using only their own knowledge, experience, and the allocated SPS guidelines and training resources without third-party interference. Thus, we are confident that we captured representative data regarding the local swine managers and veterinarians' capabilities to create SPS plans and maps at that moment in time using the available resources. Lastly, a distinctive contribution of this study is its pioneering analysis of compliance, gauged through the creation of biosecurity plans in adherence to specific guidelines. This aspect holds paramount significance, particularly in the context of prioritizing resources and efforts related to FAD preparedness. To the best of our knowledge, this study stands as the inaugural endeavor to provide evidence that actual creation of biosecurity plans in accordance with well-defined guidelines is complex and far-from-perfect in real-life conditions. This finding offers valuable insights for decision-makers tasked with optimizing resource allocation and strategic planning to enhance the nation's preparedness for potential FAD incursions.

This study is not without its limitations. First, its focus was disproportionately oriented toward large commercial sites in the area, leading to an underrepresentation of small to medium-sized producers. This skew can be partially attributed to the intrinsic nature of FAD biosecurity plans, which are encouraged to be developed in collaboration with herd veterinarians. Small to medium-scale producers commonly lack regular interactions with veterinarians, possibly contributing to their lower participation in the study. Moreover, it is plausible that this demographic, owing to the size of their operations, may have lower awareness and motivation to engage in FAD preparedness efforts, given that pork production may not constitute their primary or sole source of income. Another notable limitation pertains to the lower number of obtained written plans (compared to maps), preventing a comprehensive analysis of the full dataset for certain statistical models. The reluctance to provide written plans, often perceived as more demanding, could stem from the prioritization of other operational tasks within agricultural systems. While this limitation is acknowledged, it is important to note that the missing plans were specific to one production company. Efforts were made to address this by treating this clustering level as a random effect in our analysis. Finally, using a modeled expected feral swine density data set based on geography does not represent the true feral swine density for any given site, which may differ greatly locally across both years and seasons. Therefore, the results regarding feral swine density should be interpreted conservatively and as a theoretical guideline.

In conclusion, this study underscores the need for a rigorous evaluation of biosecurity plans in the context of FAD preparedness. The identified inconsistencies in plan design and execution highlight the importance of refining and standardizing biosecurity measures, ensuring their efficacy in mitigating the risks associated with potential disease outbreaks. Future efforts should be directed toward enhancing the comprehensiveness and alignment of biosecurity plans with the specific aim of addressing the challenges posed by FADs.

## Data availability statement

The datasets presented in this article are not readily available because data sharing non-disclosure agreements with the industry collaborators and a third party industry organization prevents us from sharing this data. Requests to access the datasets should be directed to arruda.13@osu.edu.

## Author contributions

MC: Conceptualization, Data curation, Formal analysis, Investigation, Methodology, Project administration, Software, Supervision, Validation, Visualization, Writing—original draft, Writing—review & editing. MH: Data curation, Investigation, Project administration, Writing—original draft, Writing—review & editing. KM: Methodology, Project administration, Resources, Software, Validation, Writing—original draft, Writing—review & editing. JG: Conceptualization, Formal analysis, Resources, Software, Validation, Writing—original draft, Writing—review & editing. GM: Conceptualization, Data curation, Funding acquisition, Project administration, Resources, Software, Supervision, Writing—original draft, Writing—review & editing. AA: Conceptualization, Data curation, Formal analysis, Funding acquisition, Investigation, Methodology, Project administration, Software, Supervision, Writing—original draft, Writing—review & editing.

## References

[1] Pudenz CC Schulz LL Tonsor GT. Adoption of secure pork supply plan biosecurity by US Swine producers. Front Vet Sci. (2019) 6:146. 10.3389/fvets.2019.00146 31192233 PMC6546718

[2] Hashem NM González-Bulnes A Rodriguez-Morales AJ. Animal welfare and livestock supply chain sustainability under the COVID-19 outbreak: an overview. Front Vet Sci. (2020) 7:582528. 10.3389/fvets.2020.582528 33195601 PMC7593325

[3] Hayes DJ Schulz LL Hart CE Jacobs KL. A descriptive analysis of the COVID-19 impacts on US pork, turkey, and egg markets. Agribusiness. (2021) 37:122–41. 10.1002/agr.21674 33362337 PMC7753661

[4] Picasso-Risso C Vilalta C Sanhueza JM Kikuti M Schwartz M Corzo CA. Disentangling transport movement patterns of trucks either transporting pigs or while empty within a swine production system before and during the COVID-19 epidemic. Front Vet Sci. (2023) 10:1201644. 10.3389/fvets.2023.1201644 37519995 PMC10376687

[5] Cox R Revie CW Hurnik D Sanchez J. Use of Bayesian Belief Network techniques to explore the interaction of biosecurity practices on the probability of porcine disease occurrence in Canada. Prev Vet Med. (2016) 131:20–30. 10.1016/j.prevetmed.2016.06.015 27544248 PMC7114090

[6] Gunn G Heffernan C Hall M McLeod A Hovi M. Measuring and comparing constraints to improved biosecurity amongst GB farmers, veterinarians and the auxiliary industries. Prev Vet Med. (2008) 84:310–23. 10.1016/j.prevetmed.2007.12.003 18282623

[7] Groenendaal H Costard S Zagmutt FJ Perez AM. Sleeping with the enemy: Maintaining ASF-free farms in affected areas. Front Vet Sci. (2022) 9:935350. 10.3389/fvets.2022.935350 36213391 PMC9536137

[8] Sanchez F Galvis JA Cardenas NC Corzo C Jones C Machado G. Spatiotemporal relative risk distribution of porcine reproductive and respiratory syndrome virus in the United States. Front Vet Sci. (2023) 10:1158306. 10.3389/fvets.2023.1158306 37456959 PMC10340085

[9] Bevins SN Pedersen K Lutman MW Gidlewski T Deliberto TJ. Consequences associated with the recent range expansion of nonnative feral swine. Bioscience. (2014) 64:291–9. 10.1093/biosci/biu015

[10] Lewis JS Corn JL Mayer JJ Jordan TR Farnsworth ML Burdett CL. Historical, current, and potential population size estimates of invasive wild pigs (Sus scrofa) in the United States. Biol Invasions. (2019) 21:2373–84. 10.1007/s10530-019-01983-1

[11] Bosch J Rodríguez A Iglesias I Muñoz MJ Jurado C Sánchez-Vizcaíno JM. Update on the risk of introduction of african swine fever by wild boar into disease-free European Union Countries. Transbound Emerg Dis. (2017) 64:1424–32. 10.1111/tbed.12527 27354186

[12] Jiang S-Y Ma A Ramachandran S. Negative air ions and their effects on human health and air quality improvement. Int J Mol Sci. (2018) 19:2966. 10.3390/ijms19102966 30274196 PMC6213340

[13] Alkhamis MA Arruda AG Vilalta C Morrison RB Perez AM. Surveillance of porcine reproductive and respiratory syndrome virus in the United States using risk mapping and species distribution modeling. Prev Vet Med. (2018) 150:135–42. 10.1016/j.prevetmed.2017.11.011 29169685

[14] Arruda AG Vilalta C Perez A Morrison R. Land altitude, slope, and coverage as risk factors for Porcine Reproductive and Respiratory Syndrome (PRRS) outbreaks in the United States. PLoS ONE. (2017) 12:e0172638. 10.1371/journal.pone.0172638 28414720 PMC5393554

[15] Jara M Crespo R Roberts D Chapman A Banda A Machado G. Development of a dissemination platform for spatiotemporal and phylogenetic analysis of avian infectious bronchitis virus. Front Vet Sci. (2021) 8:259. 10.3389/fvets.2021.624233 34017870 PMC8129014

[16] Hernández FA Carr AN Milleson MP Merrill HR Avery ML Parker BM. Dispersal and land cover contribute to pseudorabies virus exposure in invasive wild pigs. Ecohealth. (2020) 17:498–511. 10.1007/s10393-020-01508-6 33447876 PMC8192353

[17] Machado G Galvis JA Cardenas NC Ebling D Freeman A Hong X. The Rapid Access Biosecurity (RAB) app^*TM*^ *Handbook*. (2023). Available online at: https://osfio/p5uwq/download (accessed January 1, 2024).

[18] USDA-APHIS United States Department of Agriculture - Animal and Plant Health Inspection Service. African Swine Fever Response Plan: The Red Book. Forigen Animal Disease Preparedness & Response Plan. (2020). Available online at: https://www.aphis.usda.gov/animal_health/emergency_management/downloads/asf-responseplan.pdf (accessed January 2, 2024).

[19] Lewis JS Farnsworth ML Burdett CL Theobald DM Gray M Miller RS. Biotic and abiotic factors predicting the global distribution and population density of an invasive large mammal. Sci Rep. (2017) 7:44152. 10.1038/srep44152 28276519 PMC5343451

[20] USDA-APHIS United States Department of Agriculture -Animal and Plant Health Inspection Service. Protect our Pigs: Fight African Swine Fever. (2022). Available online at: https://www.aphis.usda.gov/aphis/resources/pests-diseases/asf/(accessed January 2, 2024).

[21] Myers LM Fromberg L. (A324) Emergency management preparedness and response planning in the US: Aphis Foreign animal disease preparedness and response plan (FAD PREP). Prehosp Disaster Med. (2011) 26:s91. 10.1017/S1049023X11003086

[22] Moore DA Merryman ML Hartman ML Klingborg DJ. Comparison of published recommendations regarding biosecurity practices for various production animal species and classes. J Am Vet Med Assoc. (2008) 233:249–56. 10.2460/javma.233.2.249 18627227

[23] Hennenfent AK Rumsey KA Pruisner RD Kaisand JJ. An overview from the 2019 swine fever exercise for agriculture response in Iowa. J Am Vet Med Assoc. (2020) 257:607–12. 10.2460/javma.257.6.607 32857009

[24] Agrawal I Bromfield C Varga C. Assessing and improving on-farm biosecurity knowledge and practices among swine producers and veterinarians through online surveys and an educational website in Illinois, United States. Front Vet Sci. (2023) 10:1167056. 10.3389/fvets.2023.1167056 37360407 PMC10289165

[25] Cadenas-Fernández E Ito S Aguilar-Vega C Sánchez-Vizcaíno JM Bosch J. The role of the Wild Boar spreading african swine fever virus in Asia: another underestimated problem. Front Vet Sci. (2022) 9:844209. 10.3389/fvets.2022.844209 35573420 PMC9093143

[26] Glišić D Milićević V Veljović L Milovanović B Kureljušić B Ðordević I. Patterns of ASFV transmission in domestic pigs in Serbia. Pathogens. (2023) 12:149. 10.3390/pathogens12010149 36678497 PMC9862985

[27] Passafaro TL Fernandes AFA Valente BD Williams NH Rosa GJM. Network analysis of swine movements in a multi-site pig production system in Iowa, USA. Prev Vet Med. (2020) 174:104856. 10.1016/j.prevetmed.2019.104856 31786406

[28] Ribbens S Dewulf J Koenen F Mintiens K De Sadeleer L de Kruif A. A survey on biosecurity and management practices in Belgian pig herds. Prev Vet Med. (2008) 83:228–41. 10.1016/j.prevetmed.2007.07.009 17850906

