# Supplementary material for: Description of swine producer biosecurity planning for foreign animal disease preparedness using the Secure Pork Supply framework
Source: Front Vet Sci. 2024 Apr 26;11:1380623. doi: 10.3389/fvets.2024.1380623 (PMC11084286; doi:10.3389/fvets.2024.1380623)
Supplement: Supplementary file 1 [file Presentation_1.pdf]

[Farm name] Enhanced Biosecurity Plan for FAD Prevention in [State]

Submission date [Date mm/dd/yyyy]

This Biosecurity Plan is based off of the Secure Pork Supply (SPS) Self-Assessment Checklist for Enhanced Pork Production Biosecurity: Animals Raised Indoors, [August 2017] and was developed using guidance from the SPS Information Manual for Enhanced Biosecurity: Animals Raised Indoors. All documents are available at www.securepork.org. In the Plan below, all items have been implemented except those indicated which will be implemented prior to requesting an animal movement permit.

Scope of Biosecurity Plan

- National Premises Identification Number (PIN): [] and production type []
- Premises Address: [STREET ADDRESS, CITY]
- Premises GPS Coordinates: [LATITUDE, LONGITUDE]
- Animals* on primary premises: [SWINE + Others] and [NUMBER OF ANIMALS]
- Other business operations on premises? [YES OR NO]
- If yes, what? [E.G. ANY CROPS]
- Secondary premises** locations: [ANY ASSOCIATED PREMISES (I.E. BOAR STUD, GILT FINISHING) WILL HAVE INDIVIDUAL SPS PLANS, AVAILABLE AS NEEDED THROUGH SYSTEM RECORDS]
    - Will be provided to Responsible Regulatory Officials if this premises is located in an FAD Control Area

*Animals that are susceptible to FMD include cattle, pigs, sheep, goats, and elk. For biosecurity guidance for dairy cattle and beef cattle, see www.securemilksupply.org and www.securebeef.org.
**Work with your State Animal Health Official to determine if separate PINs are needed for all of your associated premises. When a premises becomes infected, all premises with the same PIN number will be considered to be infected.

1. Biosecurity Manager and Written Plan

The **designated Biosecurity Manager** for this site and their contact information follows:

NAME: [FIRST CONTACT]
PHONE: [XXX-XXX-XXXX]
EMAIL: [EMAIL ADDRESS]

In the event the Biosecurity Manager is away from the site, **their designee's** contact information is:

NAME: [SECOND CONTACT- i.e, PRODUCTION MANAGER]
PHONE: [XXX-XXX-XXXX]
EMAIL: [EMAIL ADDRESS]

The Biosecurity Manager's contact information is posted [i.e, AT THE FARM SIGN-IN AREA-OFFICE].

[i.e, FARM MANAGER] has the written authority to ensure compliance with biosecurity protocols and take corrective action as needed.

[FIRST NAME] developed the site-specific biosecurity plan with the assistance of (Keep all that apply):

[LICENSED VETERINARIAN], a licensed veterinarian whose contact information is:
PHONE: [XXX-XXX-XXXX]
EMAIL: [EMAIL]

[FIRST NAME], a consultant with [ie., COMPANY NAME] whose contact information is:
PHONE: [XXX-XXX-XXXX]
EMAIL: [EMAIL ADDRESS]

The Biosecurity Manager or their designee

- [YES/NO] communicates with and/or trains individuals entering on biosecurity measures to follow;
- [YES/NO] reviews the biosecurity plan at least monthly and updates it whenever the site goes through a change affecting biosecurity;
- [YES/NO] ensures that all individuals entering the site frequently (weekly or more often) have access to a copy of the biosecurity plan;
- [YES/NO] is capable of implementing the written plan if FMD, CSF, or ASF is diagnosed in the U.S.; and
- [YES/NO] has the authority to take corrective action, as needed, when biosecurity protocols are not followed.

A labeled premises map is included at the end of this plan and can be found on the premises at: [LOCATION].

2. Training

The people listed below are trained at least [ANNUALLY, SEMIANNUALLY, OR/AND AS NEEDED] about the biosecurity measures necessary to keep an FAD out of the herd. This training is documented and available for review upon request. [FOR ALL EMPLOYEES, FARM MANAGER, SERVICE PERSONNEL]

The Biosecurity Manager(s) informs individuals entering the site of the biosecurity measures they are to follow in a language they understand. Individuals are aware of the biosecurity concepts and procedures that apply to their specific areas of responsibility. Our required training is described below.

All individuals entering must understand how to:

- [YES] Contact the Biosecurity Manager(s)
- [YES] Respect the Perimeter Buffer Area (PBA)
- [YES] Cross the Line of Separation (LOS), if required, following arrival and biosecure entry requirements.
- [YES] Perform biosecurity measures for their specific job duties

In addition, employees must:

- [YES] Undergo biosecurity training prior to starting to work at the site;
- [YES] Understand the importance of biosecurity;
- [YES] Review the entire biosecurity plan;
- [YES] Review the labeled premises map;

[YES] Know who to report to if they see someone not complying or something preventing compliance; and
[YES] Recognize the consequences for not complying with biosecurity protocols.

Communication occurs with drivers, delivery and service personnel, veterinarians, livestock transporters, and visitors through the following methods:
[METHOD] i.e. phone calls, text messages, emails, faxes
[YES/NO] a premises map highlighting the route drivers are to follow upon entering the site

3. Protecting the Pig Herd

Site Entry

Entry to the site (such as driveways) is restricted to [NUMBER] site entries and each are labeled on the premises map at the end of this plan. [SEE MAP VIEW]

- Each entry point, including unused entries, is protected with a suitable barrier consisting of [I.e., DESCRIBE BARRIERS SUCH AS GATES, ROPES, OR CABLES] to restrict entry.
- The barrier including [DESCRIBE THE BARRIER SUCH AS GATES, ROPES, OR CABLES] is in place between the residence and the hog buildings because the entry to the residence cannot be restricted.

The entry point is secured with [DESCRIBE IF LOCKED OR THE TYPE OF BARRIER WHICH RESTRICTS ACCESS].

Signs written in [LANGUAGES] are posted at the site entry that include [i.e., STOP. NO TRESPASSING. PRIVATE PROPERTY. HELP US PROTECT ANIMAL HEALTH; FILL OTHER INFORMATION].

Perimeter Buffer Area (PBA)

The Perimeter Buffer Area is labeled on the premises map at the end of this plan. The PBA is marked on-site with [DESCRIBE (E.G. FENCING AND/OR A ROPE BETWEEN MARKED POLES].

PBA Access Point(s)

Entry to the PBA is restricted to [NUMBER] controlled PBA Access Point(s) and each are labeled on the premises map at the end of this plan. The PBA Access Points are clearly marked with a suitable barrier of [i.e., A FENCE, ROPES, DOOR OR CABLES] to restrict entry.

Signs written in [LANGUAGES] are posted at all PBA Access Points that include [BOOT WASH PROCEDURE IS MANDATORY, ETC.].

All movements (animals, vehicles, equipment, people) which enter the PBA are recorded and these documents are kept in the [i.e., FARM OFFICE AND ANIMAL MOVEMENT AT CENTRAL OFFICE] and are available for review upon request.

Deliveries are made outside of the PBA at the [GIVE LOCATION: PARKING LOT, MAIN OFFICE, ETC] and this area is indicated on the premises map and signage posted at the PBA Access Point. : Downtime [HOURS]

Vehicles and equipment entering the PBA Access Points are cleaned to remove visible contamination and then disinfected at the Cleaning and Disinfection Station.

People and items crossing through the PBA Access Points follow appropriate specific biosecurity steps, described under Biosecure Entry Procedure in this plan.

Cleaning and Disinfection (C&D) Station

There is an operational, clearly marked, and equipped C&D station(s) located [GIVE LOCATION – FARM DRIVE, SITE ENTRANCE, ETC] and it is labeled on the premises map at the end of this plan. The wash pad for the C&D station is made of [PAVED, GRAVEL, PLASTIC LINED, CONCRETE] and slopes away from animal housing, feed receiving or storage areas, waterways, and on-farm traffic areas. This site uses [DRAINAGE DITCHES, BERMS, SLOPE AND LOCATION, OR OTHER PHYSICAL BARRIERS] to manage runoff from the C&D area to ensure susceptible animals are not exposed. Runoff from the C&D Station is managed following all state and local regulations.

This site has access to all the equipment and supplies needed to successfully operate the C&D Station. The SOP for the C&D process is available upon request.

The following individuals have received documented training in proper selection and use of personal protective equipment, the principles of C&D to avoid introducing an FAD virus on the site, and are able to effectively operate the C&D Station.

- [FARM MANAGER, ALL EMPLOYEES, TRUCK DRIVERS, ETC]

In the case of inclement weather (freezing temperatures, thunderstorms, high winds) when the C&D Station cannot be operated, we have the following contingency plans to ensure vehicles do not bring visible contamination onto our site:

- [i.e., NEEDS TO BE ON-SITE – AT A MINIMUM, A BACKPACK SPRAYER KEPT IN A HEATED LOCATION WITH ACCESS TO WATER].

Designated Parking Area

The designated parking area is clearly marked onsite with [CAN ADD SIGNS, NEEDS TO BE AN ACTUAL PLAN] and labeled on the premises map at the end of this plan. It is located outside of the PBA and away from animal areas.

Line of Separation (LOS)

The Line of Separation is labeled on the premises map at the end of this plan.

LOS Access Point(s)

Entry to the LOS is restricted to [NUMBER] controlled LOS Access Point(s) and each are labeled on the premises map at the end of this plan. The LOS Access Points are clearly marked with [A VARIETY OF METHODS INCLUDING, BUT NOT LIMITED TO, DOORS, GATES, SHOWERS].

Signs written in [LANGUAGES] are posted at all LOS Access Points that include [STEPS TO CLEAN AND DISINFECT -TEXT ON SIGNS]

All movements (animals, equipment, supplies, people) which cross the LOS are recorded and these documents are kept in the [LOCATION – MAY ONLY HAVE RECORDS FOR ANIMAL MOVEMENT BUT OTHER MOVEMENTS NEED TO BE RECORDED IN A FAD EVENT] and are available for review upon request.

The designated animal loading/unloading area is labeled on the premises map at the end of this plan and this is NOT used for a people entry point unless there is no alternative.

People crossing the LOS Access Points follow appropriate specific biosecurity steps, described under Biosecure Entry Procedure in this plan.

Biosecurity measures taken when food, personal items, equipment, and supplies cross the LOS include [STAYS IN OFFICE, NOT ALLOWED IN THE BARNS. NOT CLEANED, WATER BOTTLES ALLOWED INSIDE THE LOS- PLASTIC BAGS, COMBINATION OF DISINFECTANT, WIPES, AND SPRAYS].

Securing the Buildings

[FARM MANAGERS] is/are responsible for ensuring the buildings are locked. [HOW ARE THEY LOCKED, METHOD]

4. Vehicles and Equipment

Vehicles and Equipment (non-animal transport)

All vehicles and equipment not containing live animals are cleaned to remove visible contamination and effectively disinfected prior to crossing the PBA; otherwise entry is prohibited.

Concerning the sharing of equipment with other sites:

[YES/NO] Equipment is shared with other sites within the system only when necessary and steps are taken to clean and effectively disinfect equipment prior to crossing the PBA.

[YES/NO] Equipment used on this site is not shared with equipment from other sites.

Livestock Trucks/Trailers (animal transport vehicles)

All empty animal transport vehicles are cleaned and disinfected prior to arrival at the site (outgoing loads) or before animals are loaded for delivery to the site (incoming loads).

Animal transport vehicles containing animals that are not being unloaded at this site are not allowed to cross the PBA.

[YES/NO] The PBA starts at the outer edge of the chute so the truck does not enter the PBA.

[YES/NO] The PBA is relocated while animals are unloaded. After the truck leaves, the PBA is re-established and the surface is C&D. If surface is cannot be C&D, then an adequate amount of lime is applied to cover the area.

[YES/NO] Internal transport shuttles are utilized to transport animals from the edge of the PBA to the buildings' LOS Access Point.

5. Personnel

Prior to Arriving at the Site

The Biosecurity Manager ensures that everyone who needs to cross the LOS has been instructed to arrive at the site:

[YES] with a clean vehicle interior (free of all animal manure/excrement) that has not become contaminated by soiled clothes, footwear, or other items

[YES] having showered and wearing clean clothing and footwear since last contacting susceptible animals.

- [YES OR NA] For individuals that work with animals and live on-site, showering and changing into clean clothing/footwear before leaving the house is required.

- [YES OR NA] For individuals living off-site, after showering and changing into clean clothes and footwear, they must NOT contact animals, live or dead, or facilities where they are held prior to arrival at the site.

[YES] informed of the biosecurity measures they are to take once they arrive.

These individuals have a signed Employee and Visitor Arrival Agreement on file agreeing to follow our biosecure entry procedures (described below).

Entry Logbook

Everyone crossing the LOS Access Point(s) completes the entry log, which is located [LOCATION], unless they are a scheduled worker.

The entry log is monitored by [FARM MANAGER AND/OR VETERINARIAN] on the site to ensure accurate completion.

The contact information and work schedule records for all workers are maintained and posted [LOCATION].

Biosecure Entry/Exit Procedure

When entering the PBA, all individuals must:

[YES/NO] Put on disposable or disinfectable footwear

[YES/NO] Put on gloves or apply hand sanitizer

All deliveries and items entering the PBA are recorded in the [LOCATION – EX: RESPECTIVE DEPARTMENT'S DELIVERY RECORDS AVAILABLE AT THE OFFICE AND VIA PRODUCTION MANAGERS].

Vehicles and equipment entering the PBA need to be cleaned and disinfected (Vehicles are addressed in Section 6: Vehicles and Equipment).

All individuals crossing the LOS must:

[YES/NO]Shower in and shower out

[YES] Put on site-specific coveralls or clothing and footwear at the LOS Access Point.

[YES] Absolutely no street clothes not completely covered by site-specific coveralls/hats/accessories are allowed past the LOS.

[YES/NO] Wash hands, apply hand sanitizer, or put on disposable or disinfectable gloves

[YES] The same procedure is in reverse when crossing back across the LOS from the pig side of the LOS, leaving site-specific clothing or coveralls and footwear inside the LOS.

This site includes multiple pig buildings that do not have an area for employees to change into site-specific clothing. Therefore, we take the following steps:

[YES/NO/NA] Site-specific clothing or coveralls and footwear are put on and hands are washed when entering the PBA in [LOCATION];

[YES/NO/NA] Individuals walk in the PBA to each separate building;

[YES/NO/NA] The LOS is located just inside the door of a finisher building where there is not room for a bench or room to store coveralls.

The following steps are taken in each building:

[YES/NO/NA] Individuals remove footwear and cross the LOS

[YES/NO/NA] Hands are washed, OR Gloves are put on
[YES/NO/NA] Building-specific footwear is put on

[YES/NO/NA] The same procedure is in reverse when crossing back across the LOS from the pig side of the LOS.

6. Animal and Semen (if Applicable) Movement

Incoming Animals and Semen

Pigs come only from sources with documented, enhanced biosecurity practices that align with our biosecurity practices and have no current or previous evidence of the FAD viruses. Diagnostic testing of animals as requested by the Responsible Regulatory Officials is negative.

The Biosecurity Manager will ensure that any semen arriving after the FAD has been diagnosed in the U.S. will be handled as follows:

[YES/NA] Semen is purchased only from sources with documented biosecurity practices that align with our biosecurity practices.
[YES/NA] Semen has tested negative for the virus.
[YES/NA] Semen arrives in containers that can be cleaned and disinfected effectively to minimize the risk of virus contamination.
[YES/NA] The source herd documents Active Observational Surveillance for at least 7 days prior to movement of product.

The Biosecurity Manager will ensure that any semen collected after the FAD has been diagnosed in the U.S. will be handled as followed:

[YES/NA] Frozen or chilled semen is held on the site until the semen tests negative for the virus by PCR. If the animals are healthy and the semen tests negative for the virus, the semen may be shipped.
[YES/NA] Semen is transported in disposable containers or those with exteriors that can be cleaned and effectively disinfected as it crosses the LOS.
[YES/NA] The source herd must document Active Observational Surveillance for at least 7 days prior to movement of product.

Pre-movement Isolation Period-

[YES/NO] We do not accept animals from operations within an FMD Control Area.
OR
[YES/NO] We **do not move** animals to another production site with susceptible animals for at least [XX DAYS] after receiving animal from an FAD Control Area.
AND/OR
[YES/NO] We **only accept** animals from an operation in the FMD Control Area that has not received any animals from another production site with susceptible animals for at least [# OF DAYS/NA].

Contingency Plan for Interrupted Animal Movement

In the event animal movement is stopped for several weeks, this is our plan for managing animals in a biosecure manner:

[YES] Cull animals will be humanely euthanized and properly disposed of on the operation (described under Carcass Disposal below).

Housing, feed, and healthcare equipment are available for [TOTAL HERD CAPACITY] of swine for a period of [# OF DAYS, WEEKS]. A contingency plan has been established for ration formulation, transport and market of animals that are at, approaching, or have exceeded market weight.

There is enough housing and feed available for WEANED pigs [NUMBER, ADD N/A IF THIS SITE CAN NOT HOUSE WEANED PIGS] of pig for a period of [# DAYS, WEEKS].

There is enough housing and feed available for NURSERY pigs [NUMBER, ADD N/A IF THIS SITE CAN NOT HOUSE NURSERY PIGS] of pig for a period of [# DAYS, WEEKS].

[YES] Humane euthanasia equipment and supplies are available if needed.

Loading Animals

Animals leaving the site only move in one direction across the LOS at an Access Point labeled on the premises map at the end of this plan. All areas inside the LOS that become contaminated by individuals or animals loading are cleaned with detergent and effectively disinfected by trained personnel after loading is complete. The SOP for the C&D process is available upon request.

[YES/NA] The animal loading/unloading area is NOT a people entry point.
OR
[YES/NA] Individuals enter each finisher building through the one main entry that is also used for animal loading/unloading.

The following individuals have received documented training in proper selection and use of personal protective equipment, the principles of C&D to avoid introducing an FAD virus to the site, and are able to effectively C&D the loading area:

- [i.e., ALL EMPLOYEES, UNDER THE SUPERVISION AND DIRECTION OF FARM MANAGER, WHO HAS BEEN TRAINED]

7. Carcass Disposal

In an FAD outbreak, dead animals (normal mortality numbers) are disposed of by [BURIAL, INCINERATION, COMPOSTING, RENDERING, ETC – LIST ALL THAT MAY APPLY] in a way which prevents the attraction of wildlife, rodents, and other scavengers, and is in accordance with state and federal laws. Routes for carcass movement and disposal are labeled on the premises map at the end of this plan.

[YES/NO] Dead animals are disposed of within the PBA using [METHOD/NA].
[YES/NO] Dead animals are disposed of onsite but outside of the PBA.
[YES/NO] Rendering trucks and other vehicles hauling dead animals to a common disposal site do not enter the PBA.

In the event of a large number of mortalities unrelated to the FAD infection (toxicity, heat stress, etc.), dead animals will be disposed of by [LIST METHODS] which also prevents the attraction of wildlife, rodents, and other scavengers, and is in accordance with state and federal laws.

8. Manure Management

Manure is stored [LIST METHOD, IE. LAGOON, DEEP PITS, ETC]

In the event of a prolonged outbreak, we can store manure for [i.e., GIVE A RANGE, VARIABLE DEPENDING ON AMOUNT OF RAINFALL]. After that time, the method for manure removal is [LIST METHOD]. This will prevent exposure of susceptible animals and meet state, local and Responsible Regulatory Officials regulations.

[YES/NO] Site-specific dedicated manure handling equipment used within the PBA is not shared with other sites.

OR

[YES/NO] All manure handling vehicles and equipment from other sites is cleaned to remove all manure and disinfected with either heat or a chemical disinfectant followed by drying. In the event that manure handling equipment from another location cannot be effectively C&D, a plan to temporarily modify the PBA near the manure storage facility during manure removal is in place. The affected areas are cleaned and disinfected before returning to the original PBA.

All manure handling personnel must have showered and changed into clean clothes and footwear prior to arriving at the site. These expectations have been communicated to contract companies, and signed and dated when read. This communication is kept on file here: [LOCATION].

9. Rodent, Fly, Wildlife, and Other Animal Control

Rodent and Fly Control

The following rodent and fly control measures are in place

This site utilizes [METHOD] to discourage rodent movement and has designated [i.e., RESPONSIBLE PARTY SUCH AS FARM MANAGER OR DESIGNEE/NA] as the rodent control monitor responsible for implementing the rodent control plan. Bait is checked [FREQUENCY/NA] by [i.e., RESPONSIBLE PARTY SUCH AS FARM MANAGER OR DESIGNEE/NA] and replaced as needed. This protocol is in accordance with state and local regulations for controlling rodents.

OR

This site works with [LIST COMPANY/NA] and has [FORM OF DOCUMENTATION SUCH AS INVOICES OR DOCUMENTATION LOGS/NA] describing the frequency and observations by the company representative. This protocol is in accordance with state and local regulations for controlling rodents.

This operation utilizes [LIST METHOD(S)] for fly control and has designated [INDIVIDUAL'S NAME] as the fly control monitor responsible for implementing the fly control plan. This protocol is in accordance with state and local regulations for controlling flies.

Weeding and grass control is done [FREQUENCY].

The sanitation of general office areas is completed [FREQUENCY].

Trash is removed every [FREQUENCY]. In an outbreak, the garbage truck [WILL/WILL NOT] cross the PBA.

Wildlife and Other Animal Control

The following control measures are in place to minimize an animal from entering the buildings.

This operation utilizes [ie., LIST METHODS IE FENCE, BUILDING STRUCTURE TYPICAL OF CONFINEMENT REARING, WALLS] to discourage animal entry and has designated [RESPONSIBLE PARTY IE FARM MANAGER] as the individual to monitor.

[YES/NO] Dogs, cats, and other pets are NOT allowed to enter the buildings.

[YES/NO] Bird netting is used to keep birds out of the building.

[YES/NO] The buildings are totally enclosed so birds cannot enter.

10. Feed

Grain and feed commodities are delivered in trailers that are covered during transport.

In an outbreak, feed trucks delivering feedstuffs or finished feed would

[YES/NO] Feed is augered into feed bins or bagged feed is unloaded without feed trucks entering the PBA.

OR

[YES/NO] Feed trucks are cleaned and disinfected prior to entering the PBA to fill feed bins or to unload bagged feed.

Feed spills are cleaned up and disposed of as soon as possible to minimize attraction of wildlife and rodents.

Feed spills clean-up and disposal is monitored by [i.e., IE FARM MANAGER; ALL EMPLOYEES].

Labeled Premises Map

Label your premises map with the following:

- Perimeter Buffer Area (PBA)
- PBA Access Point(s)
- Line of Separation (LOS)
- LOS Access Point(s)
- Vehicle cleaning and disinfection (C&D) station(s)
- Designated parking area
- Carcass disposal/pickup location
- Carcass removal pathways
- Vehicle movements (animal transport vehicles, deliveries, etc.)
- Site Entry

INSERT MAPS WITH REQUIRED LABELING

Legend	Symbol	Legend	Symbol
LOSAE (LOS Animal Emergency)		LOSAP (LOS Access Point)	
SE (Site Entry)		CRP (Carcasses Removal Pathways)	
PBAAE (PBA Access Entry, animals only)		VM (Vehicle Movements)	
DCD (Designated Cleaning and Disinfection vehicle station)		LC (Loading Chute)	
GCD (Generalized Cleaning and Disinfection temporary station)		PBA (Perimeter Buffer Area)	
ADU (Dumpster for dead animals)		LOS (Line of Separation)	
TD (Trash Dumpster)		DPA (Designated Parking Area)	
SA (Supply drop-off Area)		CD (Carcasses Disposal location)	
PBAAP (PBA Access Point)			
